# A Bayesian dose-finding procedure applied to a seamless phase I/II trial in rheumatoid arthritis

**DOI:** 10.1186/1745-6215-12-S1-A90

**Published:** 2011-12-13

**Authors:** Anne Whitehead, Helene Thygesen, Vladimir Dragalin, John Whitehead

**Affiliations:** 1 Medical and Pharmaceutical Statistics Research Unit, Department of Mathematics and Statistics, Lancaster University, Lancaster, Lancashire, LA1 4YF, UK; 2 Aptiv Solutions, Morrisville, North Carolina, 27560, USA

## 

There is a growing interest amongst clinical investigators in the conduct of single trials combining the safety exploration of phase I with the initial investigations of efficacy usually made during phase II. This is being made increasingly possible through the use of biomarkers that show early signs of physiological changes that are associated with a therapeutic effect. Such a combined study calls for complex statistical models, able to capture the joint distribution of the safety and efficacy outcomes. Bayesian models are particularly attractive in such early phase studies because in interpreting small data sets, judicious use of investigators’ opinions becomes worthwhile.

We will describe a dose escalation procedure for a combined phase I/II clinical trial, based on a Bayesian model for the joint distribution of toxicity and efficacy (both considered binary variables) making no assumptions other than monotonicity: that is the risk of toxicity and the chance of benefit are both assumed to be non-decreasing as functions of dose level. The procedure will be discussed in the context of a placebo-controlled, sequential trial in rheumatoid arthritis, in which patients, in each stage, are randomized across all doses levels that appear safe and non-futile at the time of recruitment. The primary efficacy outcome is a binary response at 16 weeks related to an assessment known as the ACR20, but an earlier efficacy assessment based on the ACR20 assessment and reduction in C-reactive protein at 4 weeks is used during the dose escalation phase for making decisions on doses for the next cohort. The measure of safety is the occurrence of a dose limiting toxicity within 4 weeks of treatment. Based on data from a pilot study, we constructed five different scenarios for the dose-response relationships for which we simulated the trial and assessed the performance of the procedure. The new method appears to have satisfactory operational characteristics, and is flexible in that it can be adapted to the logistics of a particular trial and incorporate a placebo arm.

